# A patient with solid gynecologic cancer causing lactic acidosis, severe hypercalcemia, and hypoglycemia

**DOI:** 10.1002/ccr3.1904

**Published:** 2018-11-12

**Authors:** John Hausken, Ellen M. Haave, Håkon Haugaa, Else M. Løberg, Ulf E. Kongsgaard

**Affiliations:** ^1^ Department of Anesthesiology, Division of Emergencies and Critical Care Oslo University Hospital Oslo Norway; ^2^ Department of endocrinology Oslo University Hospital Oslo Norway; ^3^ Lovisenberg Diaconal University College Oslo Norway; ^4^ Department of Pathology Oslo University Hospital Oslo Norway; ^5^ Medical Faculty University of Oslo Oslo Norway

**Keywords:** cervical squamous cell carcinoma, hypercalcemia, hypoglycemia, insulin‐like growth factor, lactic acidosis, non–islet‐cell tumor hypoglycemia, paraneoplastic syndrome

## Abstract

Though rare in cervical cancer patients, paraneoplastic syndrome usually presents with several endocrine and hormonal symptoms. Knowledge of the pathophysiology that underlies these abnormalities is beneficial to diagnosis and treatment. An interdisciplinary approach and test analysis prior to initiating specific treatment is recommended, though prognosis appears poor in advanced cases.

## 1 INTRODUCTION

Endocrine paraneoplastic syndromes occur in patients with malignant disease and are caused by tumor production of hormones or peptides leading to metabolic derangements. These syndromes are typically detected after cancer diagnosis,^1^, ^2^ and physician knowledge about these syndromes is important for patient care.^3^ Herein, we report an unusual case of paraneoplastic syndrome with three different simultaneously occurring metabolic disturbances in a 45‐year‐old female with a solid gynecologic tumor.

## 2 CASE PRESENTATION

The previously healthy patient was admitted to her local hospital with suspected malignant disease because of a history of low back pain, genital bleeding, and weight loss of 16 kg over the previous 6 months. Her medical condition was impaired, with blurred consciousness, pale skin, and dehydration. On clinical examination, multiple lymph nodes were located in the axillary, upper lateral quadrant of the right breast, and in the inguinal regions.

Laboratory tests (Table 1) revealed severe hypercalcemia combined with acute kidney injury. Treatment to lower calcium levels was initiated with intravenous hydration, loop diuretics, calcitonin, and bisphosphonate. Parathyroid hormone (PTH) level was low, excluding primary hyperparathyroidism. Multiple myeloma with lytic bone lesions or skeletal involvement was less likely. However, the patient had a very high concentration of parathyroid hormone‐related protein (PTH‐rP) (100.3 pmol/L; normal range: <2.6 pmol/L) and a paraneoplastic syndrome was suspected. An ultrasound (US) and computed tomography (CT) scan revealed a large pelvic tumor, and upon subsequent gynecological examination, cervical cancer was suspected. Moderate dilatation of the renal pelvis and calyces was found on CT and US, though percutaneous nephrostomy was not required.

**Table 1:** Biochemical data

	At admission	Second measurement 11:00 AM	Final measurement 10:15 AM	Normal range
Glucose	1.0	5.1	7.7	4.2‐6.3 mmol/L
Insulin	ND	ND	376	<160 pmol/L
C‐peptide	ND	952	5566	300‐1480 pmol/L
Proinsulin	ND	ND	39	<36 pmol/L
IGF1	ND	ND	*Prof. Frystyk laboratory,DK*	
IGF2	ND	ND	*Prof. Frystyk laboratory,DK*	
Big‐IGF2	ND	ND	*Prof. Frystyk laboratory,DK*	
Cortisol	ND	284	ND	138‐690 nmol/L
ACTH	ND	0.2	ND	<10.2 pmol/L
pH	7.23			7.35‐7.45
pCO2	5.7			4.7‐6.0 kPa
HCO3	18.0			22‐26 mmol/L
BE	‐9.6			±3 mmol/L
Lactate	9.2			0.6‐1.4 mmol/L
Albumin	23			36‐45 g/L
Total calcium	4.65			2.15‐2.51 mmol/L
Creatinine	235			45‐90 µmol/L
PTH	1.1			1.5‐7.0 pmol/L
PTH‐rP	100.6			<2.6 pmol/L

Case Report paraneoplastic syndrome in a solid gynecological tumor. ACTH, adrenocorticotropic hormone; IGF, insulin growth factor; PTH, parathyroid hormone; PTH‐rP, Parathyroid hormone‐related protein.

**Figure 1:**
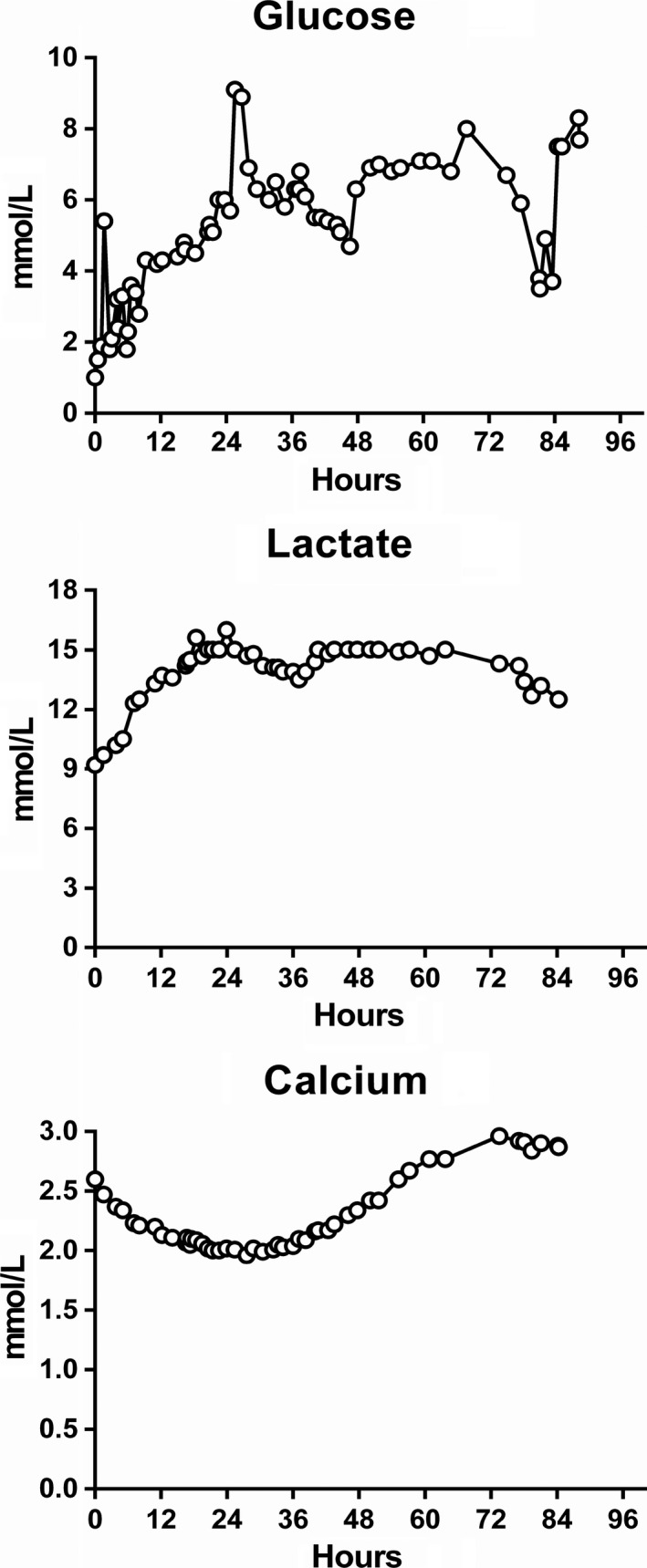
Biochemical analysis of blood‐glucose, lactate, and ionized calcium

Histopathological examinations of cervical biopsies confirmed a diagnosis of cervical squamous cell carcinoma. The patient's clinical condition deteriorated within a few days, first with worsening of her mental condition, which was already impaired at admission. Twelve days after admission, she was transferred to our tertiary referral cancer hospital for further assessment. Upon arrival, after a prolonged fasting period, she was found to have profound hypoglycemia, high lactate levels, and metabolic acidosis (Table 1). She also had sinus tachycardia (heart rate 130/min), low blood pressure (104/64 mmHg), hyperventilation (respiratory rate 30/min), SpO2 >90% (room air), and an axillary temperature of 36.7°C. Twenty mL glucose 50% bolus was administered immediately, followed by an infusion of glucose 10% at a rate of 200 mL/h. To treat the hypercalcemia, crystalloids, calcitonin (500 IU/24 hours, Miacalcic®, Essential Pharma, Surrey, UK), and glucocorticoids (dexamethasone 8 mg*4) were administered intravenously. A high urine output was targeted with furosemide 10 mg/h. Daily glucose administration was approximately 300 g to maintain acceptable blood glucose levels. Lactate and calcium levels remained elevated throughout her hospital stay (Figure 1). Since thiamine deficiency may cause lactic acidosis (LA), thiamine 100 mg was administered twice daily empirically. Reinvestigation of earlier and new biopsies from axillary lymph nodes confirmed the diagnosis of squamous cell cervical cancer. Despite referral to a critical care unit, the negative clinical course could not be reversed. Because of her advanced stage of illness, chemotherapy was considered futile. Life‐supporting therapy was therefore withdrawn, and the patient died 18 days after her first admission.

Postmortem autopsy revealed large masses of low differentiated squamous cell carcinoma filling the entire pelvis and invading the rectum and urinary bladder. In addition, peritoneal carcinomatosis and metastases to the lungs and both adrenal glands were found. No metastases were found in the brain, liver, or pancreas, but hepatic steatosis was indicated. Immunohistochemical examination with respect to insulin showed no evidence of insulin‐positive tumor cells.

Postmortem serum analyses revealed normal values of insulin‐like growth factor (IGF) 1, IGF‐2, and pro‐IGF‐2, low bioactive IGF‐2, and a high concentration of IGF‐binding protein‐2 (IGFBP‐2) (Figure 1). Furthermore, immunohistochemical analyses with anti‐IGF‐2 polyclonal rabbit antibodies (Abcam, Anti‐IGF‐2 antibody, ab9574) showed positive tumor cells, demonstrating the presence of IGF‐2‐producing cells in the primary tumor.

## 3 DISCUSSION

To our knowledge, this is the first report of a patient with gynecological malignancy simultaneously presenting three different hormonal or metabolic abnormalities: hypoglycemia, elevated lactate/LA, and hypercalcemia. Our aim in presenting this case is to evaluate paraneoplastic syndrome, possible pathophysiological mechanisms, and diagnostic challenges. As such, we do not emphasize the important oncologic perspective.

### 3.1 Hypoglycemia

Possible causes of persistent hypoglycemia in non‐diabetics include drugs, alcohol, sepsis, liver failure, hormone deficiencies (adrenal insufficiency and hypopituitarism), and endogenous hyperinsulinemia. Tumor‐induced hypoglycemia is usually related to insulin hypersecretion by a pancreatic islet tumor (insulinoma), but can also be caused by non‐pancreatic tumors called non–islet‐cell tumor hypoglycemia (NICTH).^4^ NICTH should always be considered when hypoglycemia occurs in patients with advanced malignancy,^5^ usually of mesenchymal, vascular, or epithelial cell types,^6^, ^7^, ^8^ and in patients with a large tumor burden. The most common cause of NICTH is an overproduction of IGF‐2.^9^ Daughaday et al were the first to show that tumor‐induced hypoglycemia is associated with an aberrant production of pro‐IGF‐2 (ie, big‐IGF‐2) resulting in insulin‐like activity.^10^ Plasma concentrations of IGF‐1 and IGF‐2 can be 1000 times greater than insulin in NICTH.^11^ High concentrations of IGF‐1 or big‐IGF‐2 can cause hypoglycemia by binding to insulin‐receptor, due to the homology between the IGF‐2 and insulin molecules.

Non–insulin‐dependent glucose uptake by the tumor, reflected in hyperlactacidemia, is often found in these cases and may also contribute to hypoglycemia.^12^ The metabolic alterations caused by NICTH are reversible after surgical removal of the big‐IGF‐2‐producing tumor,^13^ although metabolic disturbances usually relapse.^12^, ^14^

In the current case, neither the patient's liver enzymes, bilirubin, nor International Normalized Ratio were elevated, and she did not have any liver metastases. However, the moderate liver steatosis may have somewhat altered the hepatic capacity for glucose production. There was evidence of tumor infiltration and destruction from metastases to both adrenal glands and adrenal insufficiency with reduced cortisol production that may have led to hypoglycemia. Twenty‐four hours after admission, cortisol concentration was within the physiological range. The very low adrenocorticotropic hormone at this time is usually a result of prior administration of a high dose of intravenous dexamethasone (24 mg). This patient's surprisingly high cortisol level may have also been due to interferences of dexamethasone, or another unknown interference, in the cortisol assay (Roche Diagnostics, 1st generation assay).

Unfortunately, relevant blood samples for hormone analysis were not collected at admission, when the patient had hypoglycemia, making the results more difficult to interpret. C‐peptide, insulin, and proinsulin were measured 4 days after admission (approximately 7 hours before she died), and at that time, blood glucose was within the normal range (Figure 1). Insulin and especially C‐peptide concentrations were very high, whereas proinsulin was low compared with insulin concentration. Such high levels of C‐peptide may be due to hyperglycemia or insulin resistance. Additionally, increasing renal failure in the terminal phase may have contributed to the reduced clearance of C‐peptide. Moreover, interference in the C‐peptide assay (Roche diagnostics) cannot be entirely excluded.

Since blood tests were not collected while the patient was hypoglycemic, we cannot determine if there was endogenous insulin production; however, it is unlikely that the patient had insulinoma in addition to the primary cancer. No tumors were detected in the pancreas on the CT scan or autopsy, and immunohistochemical examination of the biopsies did not detect insulin‐producing cells.

**Figure 2:**
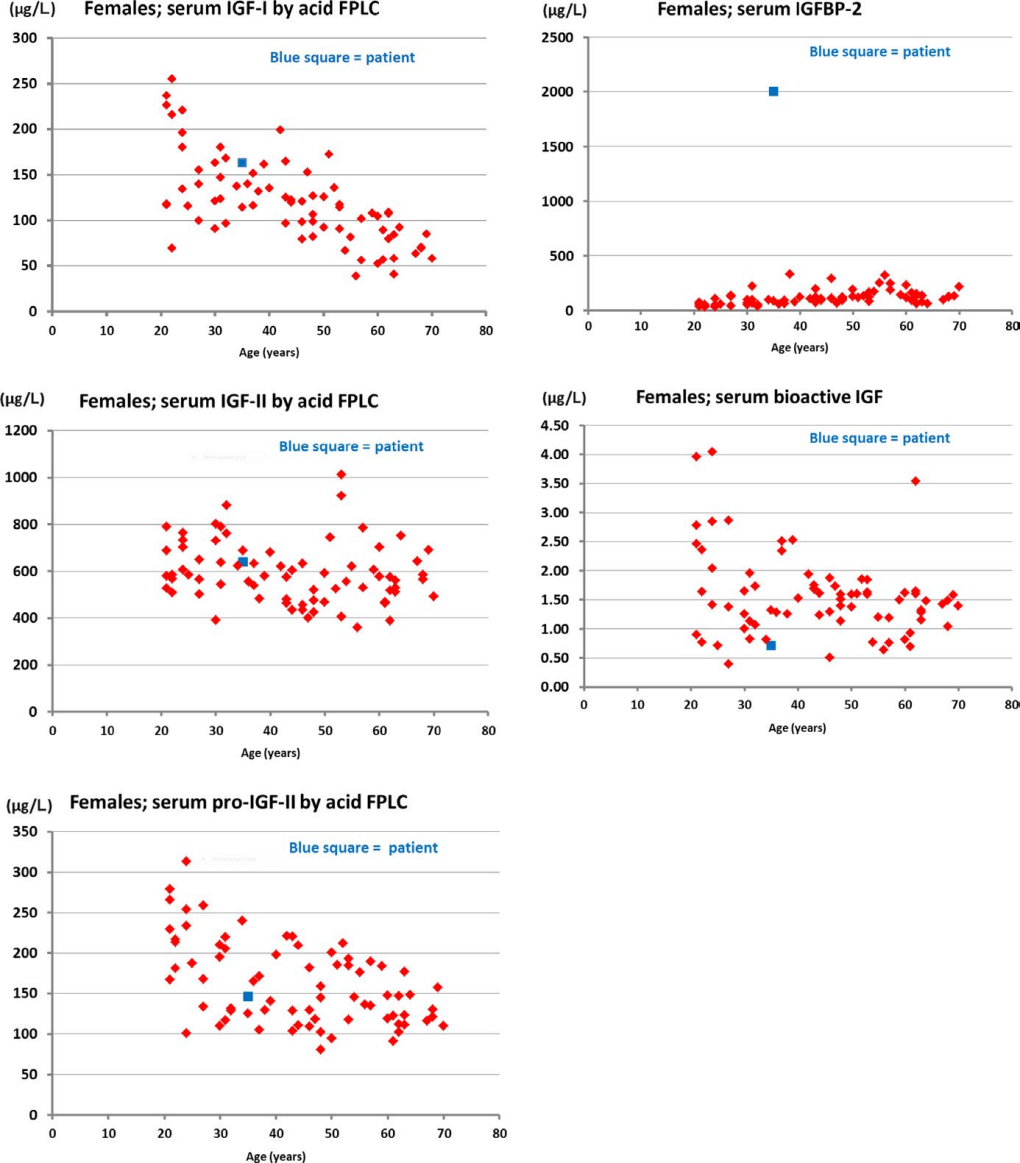
Data on serum levels of IGF‐I, IGF‐II, pro‐IGF‐II, IGFBP‐2 and bioactive IGF. Normal reference values in red, patient measurements in blue. Very high levels of IGFBP‐2 are seen in tumor‐hypoglycemia, but also in other malignant diseases. Thus, an elevated serum IGFBP‐2 is not diagnostic for tumor‐hypoglycemia. Serum IGF‐I is usually very low in patients with tumor‐hypoglycemia and this is not the case in the present patient (Frystyk et al Diabetologia 1998)

Typical findings in true cases of NICTH causing hypoglycemia include high IGF‐2, and more frequently, high IGF‐2 and low IGF‐1, with an IGF‐2/IGF‐1 ratio >10:1.^5^ Our patient had normal concentrations of IGF‐2 and pro‐IGF‐2, low bioactive IGF‐2 and high IGFBP‐2 (Figure 2). In addition, IGF1 was in the normal range. High levels of IGFBP‐2 are seen in tumor‐hypoglycemia, but also in other malignant diseases, thus an elevated serum IGFBP‐2 is not diagnostic for tumor‐hypoglycemia.^15^ Unfortunately, no samples were taken during hypoglycemia, which complicated the interpretation. In addition, samples were taken several days after starting high‐dose steroid treatment, which may have attenuated the typical findings.

**Figure 3:**
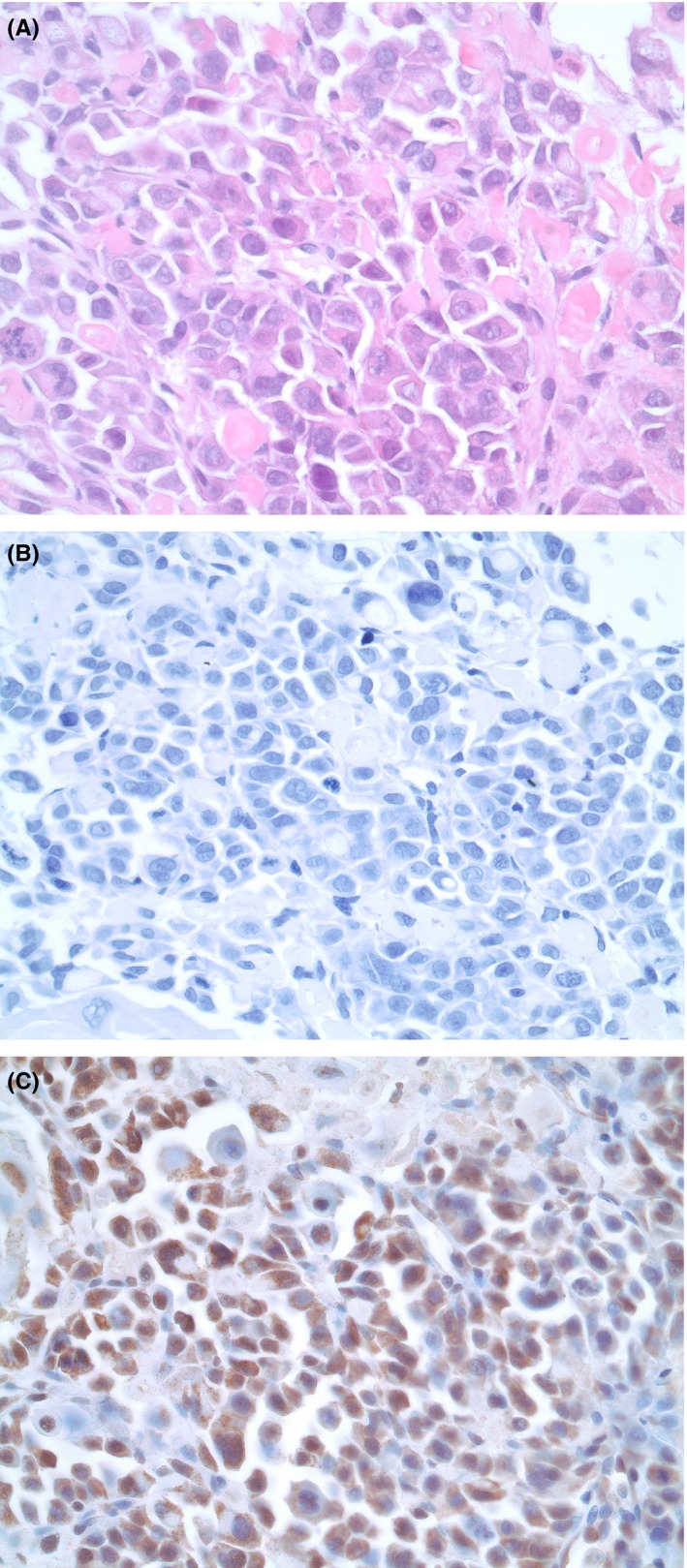
Biopsy from an axillary lymph node with metastasis of a poorly differentiated squamous cell carcinoma. A, Hematoxylin and eosin (H&E) staining, obj. ×40. B, Immunohistochemical staining with anti‐insulin antibody, obj. ×40. The tumor cells are negative. C, Immunohistochemical staining with anti‐IGF2 antibody, obj. ×40. The tumor cells are positive (brown)

Steroids decrease and suppress big‐IGF‐2,^7^, ^12^, ^16^ and large doses may provide remarkable improvements in glucose homeostasis and other biochemical markers of IGF‐2‐producing tumors.^17^, ^18^ This is probably the reason for such findings in our patient, for whom laboratory analyses of IGF‐1 and IGF‐2/big‐IGF‐2 were completed several hours after administration of steroids. However, immunohistochemical staining with anti‐IGF‐2 was positive, most likely proving the presence of IGF‐2‐producing cells in the primary tumor, which caused severe hypoglycemia (Figure 3).

IGFs are multifunctional peptides that play a pivotal role in cell regulation; they consist of IGF‐1 and IGF‐2, with three cell‐membrane receptors and IGF‐binding proteins. These factors can play an important paracrine/autocrine role in promoting tumor growth during tumor progression; however, these roles vary depending on the tissue origin.^19^, ^20^, ^21^ IGF‐1 may also be a useful biomarker for assessing risk and early diagnosis of precancerous growth in the cervix,^22^ and IGF‐2 may be a reliable marker for early diagnosis and the monitoring of therapy efficacy.^23^

### 3.2 Lactic acidosis (LA)

Hyperlactatemia is defined as a mild‐to‐moderate (2‐4 mmol/L) increase in blood lactate concentration without metabolic acidosis, whereas LA is characterized by persistently increased blood lactate levels (usually >5 mmol/L) associated with metabolic acidosis. LA is divided into two subtypes: type A and B. Type A is due to tissue hypoxia, whereas type B occurs without any evidence of poor tissue perfusion or oxygenation.^24^ However, LA can also be a rare complication in solid tumors, as first reported in acute leukemia by Field et al .^25^ The mechanism behind this is incompletely understood and probably multifactorial, but may be partly explained by an enhanced aerobic glycolytic activity in cancer cells .^26^ In contrast to normal tissue, cancer cells frequently develop a modified glucose metabolism (Warburg effect), whereby blood glucose consumed by the tumor cells is converted to lactate even in the presence of adequate oxygen.^27^, ^28^ In our case, despite evidence of hepatic steatosis, liver failure could be biochemically excluded as a possible cause of tissue hypoperfusion because hypotension or sepsis was most likely not the case. LA type B implies a poor prognosis; in a review of 29 cases, 22 died within a few weeks after its detection,^26^ and patients whose disease was not treated or who did not respond to chemotherapy died with active LA.^29^ In our patient, administration of glucose suggested increased lactate production by the tumor.

### 3.3 Hypercalcemia

All types of malignant neoplasms can cause hypercalcemia, which is divided into four types^30^: (a) increased secretion of PTH‐rP from tumor cells, (b) osteolytic activity of bone metastases, (c) hypercalcemia due to elevated secretion of vitamin D, and (d) ectopic secretion of PTH from the tumor.

Production of PTH‐rP is a common cause of hypercalcemia and is the most common paraneoplastic endocrine syndrome.^31^ About 80% of patients with malignancy‐associated hypercalcemia (MAH) have increased PTH‐rP serum levels,^32^ and the high calcium levels are a result of the combined effects of PTH‐rP on kidney and bone. Secretion of endogenous PTH is suppressed by PTH‐rP‐mediated hypercalcemia, as was the case in our patient. Clinical manifestations of hypercalcemia include nausea/vomiting, weakness, and altered mental status, and may provoke life‐threatening arrhythmias when calcium levels exceed >4 mmol/L. When MAH occurs in patients with advanced cancer,^32^ life expectancy is poor; a case series found that median survival is only 30 days.^33^

## 4 CONCLUSION

The combination of hypoglycemia with LA and hypercalcemia is uncommon and, to our knowledge, has not been previously reported in a patient with a solid gynecologic tumor. Exploring the mechanisms behind a paraneoplastic syndrome may be challenging. Methodical interpretation of laboratory findings and involvement of an interdisciplinary team of clinicians are both valuable. Since glucocorticoid therapy may alter the biochemical picture, we recommend postponing its administration until all necessary laboratory samples have been obtained.

## CONFLICT OF INTEREST

The authors declare that they have no competing interests.

## AUTHORS’ CONTRIBUTIONS

All authors contributed to writing and revising the manuscript. The authors are experienced anesthesiologists, endocrinologist and pathologists involved in the multidisciplinary management of cancer patients at the comprehensive cancer center of a large tertiary care university hospital.

## CONSENT FOR PUBLICATION

A copy of the written consent is available for review by the journal editor.

## AVAILABILITY OF DATA AND MATERIAL

Data are available in the hospital's medical records.
